# Biomechanical and Physiological Evaluation of a Multi-Joint Exoskeleton with Active-Passive Assistance for Walking

**DOI:** 10.3390/bios11100393

**Published:** 2021-10-15

**Authors:** Wujing Cao, Zhewen Zhang, Chunjie Chen, Yong He, Dashuai Wang, Xinyu Wu

**Affiliations:** 1 Guangdong Provincial Key Lab of Robotics and Intelligent System, Shenzhen Institute of Advanced Technology, Chinese Academy of Sciences, Shenzhen 518005, China; wj.cao@siat.ac.cn (W.C.); cj.chen@siat.ac.cn (C.C.); yong.he@siat.ac.cn (Y.H.); xy.wu@siat.ac.cn (X.W.); 2 SIAT Branch, Shenzhen Institute of Artificial Intelligence and Robotics for Society, Shenzhen 518005, China; 3 CAS Key Laboratory of Human-Machine Intelligence-Synergy Systems, Shenzhen Institute of Advanced Technology, Shenzhen 518005, China; 4 Guangdong-Hong Kong-Macao Joint Laboratory of Human-Machine Intelligence-Synergy Systems Rehabilitation Engineering and Technology Institute, Shenzhen 518005, China; 5 Rehabilitation Engineering and Technology Institute, University of Shanghai for Science and Technology, Shanghai 200093, China; zhangzw@siat.ac.cn

**Keywords:** lower limb exoskeleton, muscle activity, metabolic cost, active-passive assistance, force tracking

## Abstract

How to improve the walking efficiency while ensuring the wearability is an important issue of lower limb exoskeletons. Active devices can provide greater forces, while the passive devices have advantage in weight. We presented a multi-joint exoskeleton with active hip extension assistance and passive ankle plantarflexion assistance in this work. An admittance controller based on a feedforward model was proposed to track the desired active force of the hip extension. An underfoot clutch mechanism was adapted to realize the passive ankle plantarflexion assistance. To assess the efficacy of the multi-joint exoskeleton in assisting walking, we conducted comprehensive experiments to evaluate the force tracking performance, lower limb muscle activities and metabolic cost. The results demonstrated that: (i) The average tracking error of the peak hip extension assistance force from three subjects was less than 3%. (ii) The reductions of normalized root-mean-square EMG in the lateral soleus, medial soleus and gluteus maximus of eight subjects achieved 15.33%, 11.11%, and 3.74%, respectively. (iii) The average metabolic cost of six subjects was reduced by 10.41% under exoskeleton on (EO) condition comparing to the condition of walking with no exoskeleton (NE). This work proved that the concept of the multi-joint exoskeleton with active-passive assistance can improve the walking efficiency.

## 1. Introduction

Recently, various lower limb exoskeletons (LLEs) have been proposed for aiding people with lower limb motor dysfunction, soldiers, and workers [1,2,3,4]. The LLEs are mainly divided into three categories according to the used materials: rigid LLEs made up of hard linkages, soft-driven LLEs which have soft actuators and rigid frame and joints, and soft LLEs without rigid frames and joints but consist of garments, cables or straps [5]. A high and precise force can be obtained by using a rigid LLE, but its large stiffness limits the natural motion of a wearer [6,7] and the misalignment of the biological joint may cause secondary injury to a patient [8]. On the other hand, the soft-driven and soft LLEs allow wearers to walk freely due to the flexibility of the used materials and actuators. However, the soft LLEs cannot provide support assistance during loaded walking due to their soft components. Reducing walking effort and improving efficiency are the focus of much research on soft LLEs.

Active and passive soft LLEs have been introduced for reducing the walking burden [9,10]. The metabolic cost and muscle activity are commonly used as quantitative metrics to assess whether walking effort is reduced [11]. Mooney et al. assessed the reduction of metabolic cost of an active soft-driven ankle exoskeleton in a load condition [12]. Jackson et al. evaluated the performance of a soft-driven ankle exoskeleton by the normalized root-mean-square (RMS) of the soleus muscle activity and metabolic rates [13]. Zhang et al. developed a human-in-the-loop method for identifying the assistance of the exoskeleton which was proposed to minimize the metabolic cost during walking [14] and compared the lower limb muscle activities under different assisting conditions [15]. However, the testing conditions presented in [13,14,15] were tethered. It is worth noting that the added distal mass may be a major factor that limits the walking efficiency of an active soft-driven ankle exoskeleton in wearing condition.

Since the mass added to the waist and thigh is substantially less detrimental than that added to the foot, the mass penalty will be reduced by augmenting a proximal joint [16]. Kim et al. [17] proposed a portable soft exosuit with hip extension assistance to reduce the metabolic rate of walking and running. Active soft exosuits with multi-joint assistance were also developed and evaluated by the team of Walsh et al. [18,19]. The soft exosuits proposed in [17,18,19] weighs 5–9.3 kg for portable conditions. We developed a soft exosuit with active assistance to the hip and knee [20]. However, the system weight was still 6.6 kg. The passive assistance is considered to be an important direction for reducing the added weight of LLEs and saving energy. Collins et al. [21] presented a passive ankle exoskeleton to provide ankle plantar flexion assistance by the spring attached to a mechanical clutch. Both mid-stance soleus electrical activity and metabolic cost were reduced in the weight range of 0.816–1.006 kg. Inspired by their design, Yandell et al. [22] proposed a low profile, unpowered ankle exoskeleton which is to be worn under clothes. The main difference was that the clutch was under-the-foot. The passive hip assistance exosuits were also studied by Nasiri et al. [23] and Simpson et al. [24]. Although passive LLEs have advantages in terms of weight and working time, they usually work in a specific gait and cannot be actively controlled.

**Figure 1:**
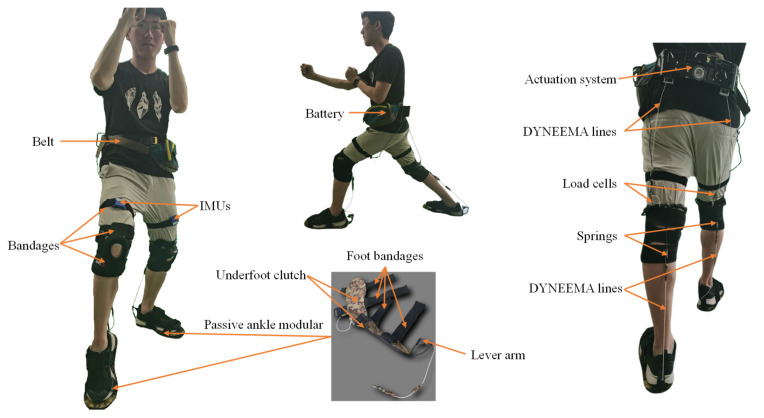
The mechanism and prototype of the proposed soft lower limb exoskeleton.

To combine the advantages of active and passive assistance, we proposed a multi-joint exoskeleton with active-passive assistance for walking. The mechanism and prototype are shown in Figure 1. The hip extension was assisted by an active actuator and passive under-foot clutch mechanism was adopted to assist ankle plantar flexion.

The major contributions of this work are as follows:

(1) In order to simultaneously solve the portability problem of the multiple motion assistance exoskeleton and break through the efficiency limitation of the single motion assistance exoskeleton, a portable soft LLE with active hip extension assistance and passive ankle plantar flexion assistance was proposed;
(2) An admittance controller based on a feed-forward model was introduced to accurately track the desired active force of the hip extension;
(3) The muscle activities (the lateral soleus, medial soleus, gluteus maximus) were analyzed by normalized RMS EMG and net metabolic cost comparison between NE and EO walking was conducted, by which the effect of the proposed LLE with active-passive assistance was evaluated quantitatively.

## 2. Materials and Methods

### 2.1. System Overview

**Figure 2:**
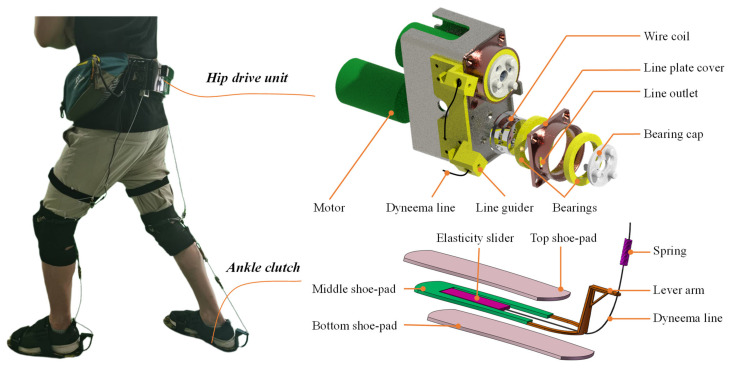
Detailed structure of the hip drive unit and passive ankle clutch.

The key aspects of our prototype include two parts, active hip module and passive ankle module, as shown in Figure 2. The active hip module is mainly composed of a belt, thigh and knee straps, an actuator unit, a battery, a sensory and a control system. The actuator is fixed on the belt. Two drive units are placed on the right and left sides of the actuator. Each drive unit includes a high power and low inertial motor (M2006, DJI, China) connected to a planetary gear reducer which is committed to driving a wire coil. The hip extension assistance forces from two drive units are transmitted to the thighs by two Dyneema lines. The active assistance forces are measured by two load cells (ZNLBS-v1, Chino Sensor, Bengbu, China) placed between the Dyneema lines and the anchor points on the straps around the knee (Figure 1). Two IMUs (LPMS-B2, Alubi, China), attached to the straps in front of the thighs (Figure 1), are used to detect the gait phase.

The passive ankle module includes ankle clutch and assistance spring. The assistance spring is parallel to the calf and serves as an artificial Achilles tendon. The Dyneema line is connected to the spring and pulls the ankle in specific gait phase through a lever arm on the back side of the heel. The bandages are placed around the knee. The location made it suitable for wearing over the pants. As shown in Figure 2, the underfoot clutch consists of three shoe-pads, an elasticity slider connected to the middle shoe-pad and the Dyneema line, a lever arm used to guide the line, and the spring connecting the heel and knee wraps. The top and bottom shoe-pads were commercialized rubber insoles. The middle shoe-pad was reshaped by cutting the rubber insole. The elasticity slider was made up of an elastic cloth of a low stiffness. One end of the elasticity slider was connected to the middle shoe-pad by super glue, and the other end is connected to the Dyneema line. The lever arm was used to amplify the ankle assistance torque generated by the spring. The total mass of the multi-joint soft exoskeleton, including the power hip module and the passive ankle module, is 3.5 kg. The hip drive module and the battery, which comprise most of the total mass, are placed close to the center of mass of the wearer.

### 2.2. Walking Assistance Strategy

**Figure 3:**
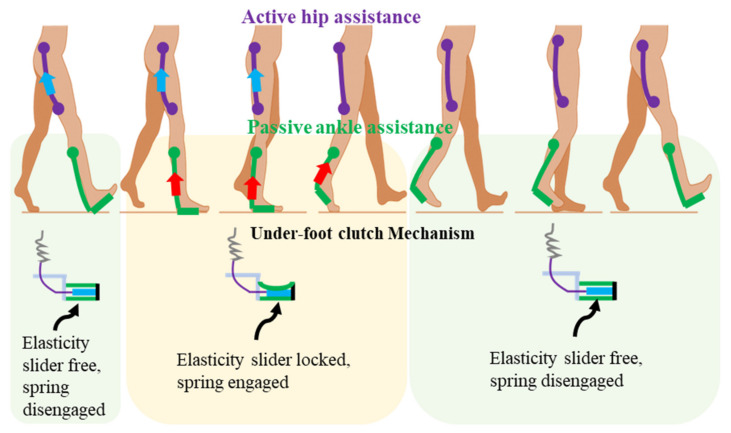
The specific assistance gait phases and realization methods.

The walking assistance strategy in this work is to assist the hip actively and the ankle passively. The first design question was: which gait phases should the exoskeleton assist? In order to improve the walking efficiency, the exoskeleton should assist those gait phases which need positive mechanical power of biological joints. The specific assistance gait phases and realization methods are shown in Figure 3. For active hip assistance, the Dyneema line connecting the thigh attachment and the actuation system is stretched during the hip extension. For the passive ankle assistance, when no force is acting on the middle shoe-pads during the leg swing and early stance, the elastic slider is free. The spring has little effect on walking. When the force is applied to the shoe-pads due to body weight from heel flat to heel off, the elasticity slider is fixed. The spring is stretched and stores energy since the motion of shank changes from vertical position to inclined posture. The stored energy is released and assists the ankle plantar flexion from the mid stance to the heel off.

The determination of the assistance strategy is shown in the experiment result 3.1. The motion capture system (VICON, Oxford, UK) with six cameras and a force measuring platform embedded in the treadmill (AMTI, Watertown, MA, USA) were used. The tests were performed by instructing the subjects to walk on the treadmill at a speed of 1.5 m/s.

### 2.3. Gait Cycle Time Prediction

To provide active hip extension assistance, the first step was to predict the gait cycle time and scale the gait to obtain the typical gait phase. The gait cycle time was obtained by two IMUs fixed on the thigh. The sample frequency of the IMU is 400 Hz. Both angle and angular velocity of hip extension were set to be positive, and the flexion was set to be negative. The difference between the angles of the left and right hip joints was used as the feature point. In this work, the feature angle of hip was calculated as follows:

(1) $$\theta_{f}=\theta_{Rh}-\theta_{Lh}$$

where $\theta_{f}$ is the feature angle of hip, $\theta_{Rh}$ is the hip angle of right side, $\theta_{Lh}$ is the hip angle of left side. The value of feature angle was chosen to be zero. The sign, positive or negative, of the angular velocity of the feature angle determines whether the next motion will be of the hip extension or that of the hip flexion. A set of coefficients are used in the Markov chain to predict the length of the current gait cycle. The prediction method is as given below:

(2) $$T_{i}=0.7\times T_{i-1}+0.2\times T_{i-2}+0.05\times T_{i-3}+0.03\times T_{i-4}+0.02\times T_{i-5}$$

where $T_{i}$ is the time between two consecutive feature angles, $T_{i-1}$, $T_{i-2}$, $T_{i-3}$, $T_{i-4}$, and $T_{i-5}$ are consecutive periods of time in the previous gait phase. A complete gait cycle was calculated as follows:

(3) $$T=T_{i}+T_{i-1}$$

where T is a complete gait cycle time.

### 2.4. Control Strategy of the Active Hip Extension Assistance

**Figure 4:**
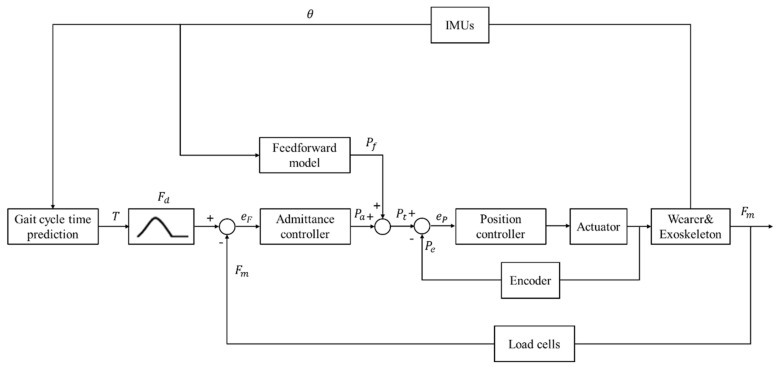
The control strategy of the active hip assistance. T: predicted gait cycle time, $F_{d}$: desired force, $F_{m}$: measured force, $e_{F}$: force error, $P_{a}$: output position of the admittance controller, $P_{f}$: output position of the feedforward model, $P_{t}$: target position of the motor, $P_{e}$: measured position of the motor, $e_{P}$: position error, θ: hip angle measured by IMUs.

When the gait cycle time is defined, the desired force was applied to the gait cycle by scale of the gait. The desired force was the same as our previous work about hip extension assistance [1]. We have proposed an iterative learning control strategy with feedforward model for a soft exoskeleton with hip and knee joint assistance [25]. Admittance control has shown good performance in the single motion assistance of soft hip exoskeleton [26]. In Figure 4, we present an admittance controller based on the feedforward model.

The control on the two legs is commanded by two equal subsystems. For simplicity, we present the design of the controller on one side only. Applying the prediction method described in Section 2.3, the gait cycle time T was predicted by hip angles θ measured by IMUs. The desired force $F_{d}$ was obtained by the scaling of the gait. The method used for establishing the feedforward model was the same as the one used in our previous work [25]. The feedforward model was added to reduce the systematic error. The equation of the feedforward model is given below:

(4) $$P_{f}=a\theta +b$$

where $P_{f}$ is the output position determined by the feedforward model, and a and b are corresponding coefficients, θ is the hip angle.

The error $e_{F}$ between the desired force $F_{d}$ and the measured force $F_{m}$ was the input to the admittance controller for calculating the terminal position $P_{a}$ of the system. The expression of the admittance control is defined in the Laplace domain as follows:

(5) $$Y=\frac{p\left(s\right)}{e\left(s\right)}=\frac{1}{M*s+C}$$

where Y is the virtual admittance, M is the virtual inertia, C is the stiffness. Target position $P_{t}$ of the motor was obtained by $P_{f}$ and $P_{a}$. The error $e_{P}$ between the target position $P_{t}$ and the position measured by motor encoder $P_{e}$ by motor encoder was the input to the position controller.

### 2.5. Materials and Methods for Assistance Force Evaluation

The spring stiffness of the passive ankle clutch is 11.18 N/mm (AUT12-45, MISUMI, Tokyo, Japan). The safe load of the spring is 120 N. The maximum lever arm length of the ankle joint was 0.12 m, including the length of the exoskeleton lever arm and the biological ankle. Since there was no actuation or sensor component in the ankle assistance mechanism, another two load cells were added on the Dyneema lines of the left and right ankles to measure the plantarflexion assistance force of the passive mechanism. The hip force is measured by the load cells placed between the Dyneema lines and the attachment points on the straps around the knee. Although the active and passive assistance forces were delivered and measured for both sides (right and left), the results were presented based on one side since the symmetry of walking. The left side was chosen to be the presenting side.

### 2.6. Materials and Methods for Muscle Activity Evaluation

The experimental process referred to the procedure presented in [15]. A training session and a testing session were set with a break of two days for each subject to avoid fatigue effects. During the training session, the subjects were acclimated to the multi-joint soft exoskeleton. In the testing session, the subjects walked on the treadmill for 7 min at a speed of 5 km/h. The first three minutes were for warming-up. The data were collected in the following three minutes as steady state data for calculating the muscle activity. The last one minute was for slowing down and stopping the walk. In the testing session, the time interval between the two test conditions (i.e., normal and assisted walking) was thirty minutes. For normal walking, the subjects performed the task without wearing the device. Under the assisted condition, the exoskeleton provided assistance only to the right lower limb.

The Biometrics PS850 system (Biometrics, Britain) was used to collect the EMG signals. The sample frequency was 2000 Hz. The raw EMG signals were processed by a high-pass filtered with a second-order Butterworth filter (frequency 10 Hz), low-pass filtered with a second-order Butterworth filter (frequency 250 Hz), and notch filter (frequency 50 Hz). Afterwards, we sorted out the data and conducted statistical analysis in MATLAB (MathWorks, Natick, MA, USA). All the preprocessed EMG amplitudes were then normalized to the average EMG peak value during the steady walking state for the same participants.

**Figure 5:**
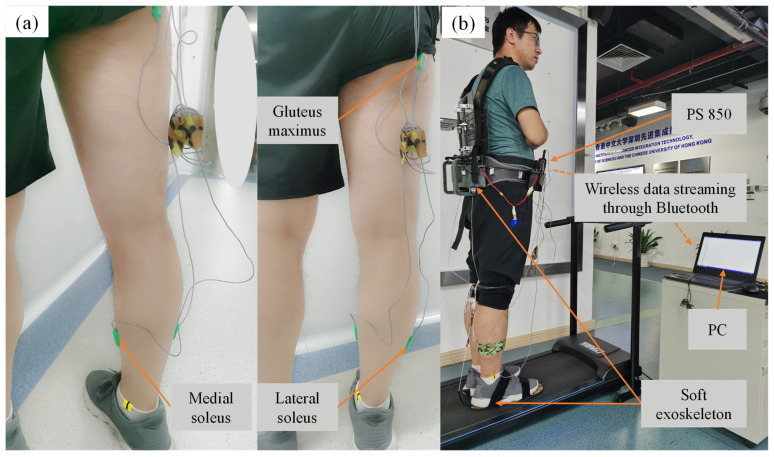
The position of the EMG electrodes on the leg and test scheme. (**a**) EMG electrodes on the leg, (**b**) test scheme.

The position of the EMG electrodes on the leg and the test scheme are shown in Figure 5. Three right lower limb muscles (gluteus maximus, lateral soleus and medial soleus) were selected for collecting the EMG signals. The reason was that lateral soleus and medial soleus were mainly responsible for the ankle plantarflexion in the sagittal plane. It is a general approach to measure the responsible muscle for the joint motion. The references [15,22] provided ankle exoskeletons for plantarflexion assistance. They also measured the EMG of the same muscles with our work.

### 2.7. Materials and Methods for Metabolic Cost Evaluation

The testing protocol was the same as our previous work [1]. The K5 mask (Cosmed, Italy) was used to measure the volumetric oxygen consumption and carbon dioxide expulsion rates. Two walking conditions includes no exoskeleton (NE) and exoskeleton on (EO) were compared. The net metabolic rate calculation and statistical analysis methods were detailed in our previous work [1]. The final results of case are reported in the form of the mean ± standard error of the mean (SEM).

## 3. Results

All the subjects are healthy and have signed an informed consent prior to their participation in the following experiments and all the experiments were approved by the authors’ Institutional Review Board. The subjects are different on each experiment.

### 3.1. The Determination of Assistance Strategy

The assistance phases of the hip and ankle were determined by biological joints power test. Ten subjects (8 men and 2 women; average age 25, range 23–29) were recruited on a volunteer basis for the acquisition of biological data. The experimental procedures were clearly explained to the subjects and their consents were obtained prior to the enrollment for the study.

**Figure 6:**
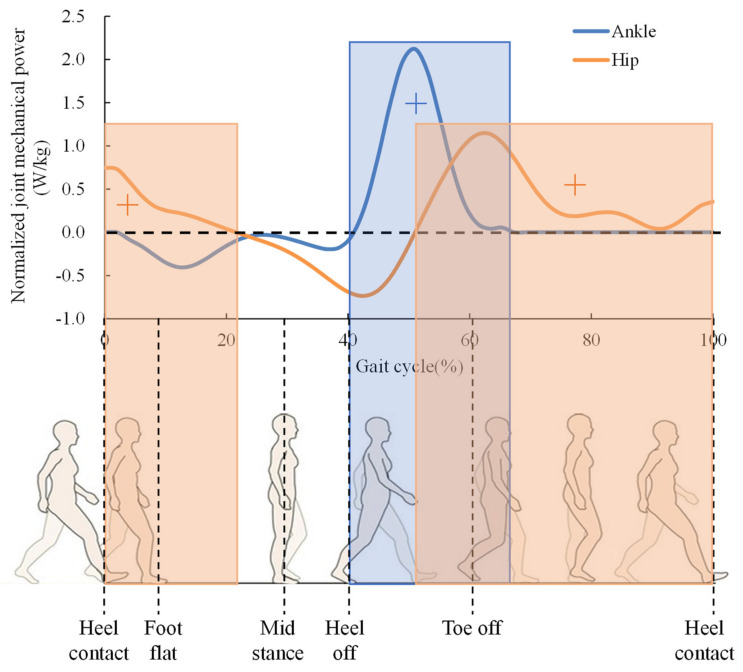
The mechanical power of the hip and ankle during walking. The orange areas indicate the phases need positive work of hip joint. The light blue areas indicate the phase need positive work of ankle joint.

Figure 6 shows the mechanical power of the hip and ankle during walking. The phases of hip, requiring positive power during walking, are about 0~20% and 50~100% of the gait cycle. The corresponding motions are the hip extension during stance and the hip flexion during swing. Therefore, assisting both the hip extension and flexion can be more beneficial for improving the walking efficiency. However, we should consider the balance of the system weight and the complexity of the mechanical structure. Since the actuation system is placed on the back side, the leg joint motion is easily driven in the rear direction than in the forward direction. Hence, the hip extension during the stance phase is chosen to be assisted actively. The ankle joint requires positive power mainly from 40% to 66% of the gait cycle. During the plantar flexion, the ankle joint produces a peak of positive power. The ankle joint requires more amount of positive power than the hip joint and, in addition, it is also more distal than the hip. Preliminary studies have indicated that when mass is added to the extremities, the metabolic rate increases disproportionately with the load mass and for more distal load location [27,28]. These findings suggest that the mass added to the foot of the wearable exoskeleton should be minimized. Therefore, the passive assistance mechanism was adopted to assist the ankle plantar flexion. As a result, the walking assistance strategy of the soft exoskeleton provides active assistance for the hip extension and passive assistance for the ankle plantar flexion during the stance phase.

### 3.2. Test of the Gait Cycle Length Prediction Method

A male was recruited to test the prediction method of the gait cycle length. The subject was asked to walk comfortably.

**Figure 7:**
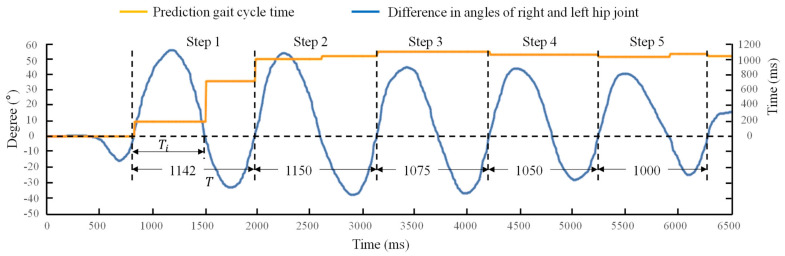
The difference in angles of right and left hip joint and prediction gait cycle time. $T_{i}$ is the time between two feature angles, T is a complete gait cycle time.

The measured difference between the angles of the right and left hip joints and the predicted gait cycle time are shown in Figure 7. A small jitter was observed before the first step. It means that the wearer lifted his leg slightly forward before starting normal walking. In Step 1, the prediction of the gait cycle time was far away from the actual time. However, the error was reduced in the second time interval between the two zero difference angles of Step 1. The prediction of the gait cycle time is accurate from Step 2. The same results were found in Steps 3, 4 and 5 also. The results showed that the method is suitable to predict the gait cycle time. When the gait cycle time is defined, the desired force was applied to the gait cycle by scale of the gait.

### 3.3. Assistance Force Evaluation

The assistance force exerted by the exoskeleton were tested by three healthy male subjects (age 24.3 ± 2.0 years, mass 68.8 ± 3.8 kg, height 1.73 ± 0.04 m, mean ± SD). The subjects were asked to walk on the treadmill at a speed of 5 km/h. Each trial lasted five minutes.

**Figure 8:**
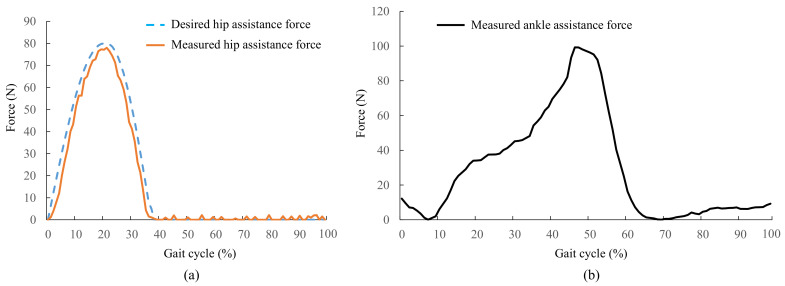
Active-passive assistance force. (**a**) Force tracking performance of active hip extension assistance; (**b**) the passive ankle assistance force.

The mean values of the desired hip assistance force and the measured hip assistance force in fifteen sequential cycles with the admittance controller based on the feedforward model are shown in Figure 8a. The peak time of the hip assistance force was at 21.5±1.9% of the gait cycle, where the desired peak time was 22%. There was a little delay and no overshoot of the peak force. At the time of the desired peak of the hip assistance force, the measured hip extension force averaged between the three subjects was 78.2 N, whereas the desired peak force was 80 N. The error in the peak force was 2.25%. This small error might have been caused by the insufficient tracking of the position controller. The measured intermittent small force during the late-stance and swing phase was likely due to the inadequate coupling between the load cell and the actuator of the exoskeleton.

The passive ankle assistance force is shown in Figure 8b. The peak assistance force of the ankle was 99.3 N. The assistance time covered the plantarflexion phase of the ankle. The trend of the ankle assistance force was consistent with the natural ankle plantar flexion torque.

### 3.4. Muscle Activity Evaluation

**Figure 9:**
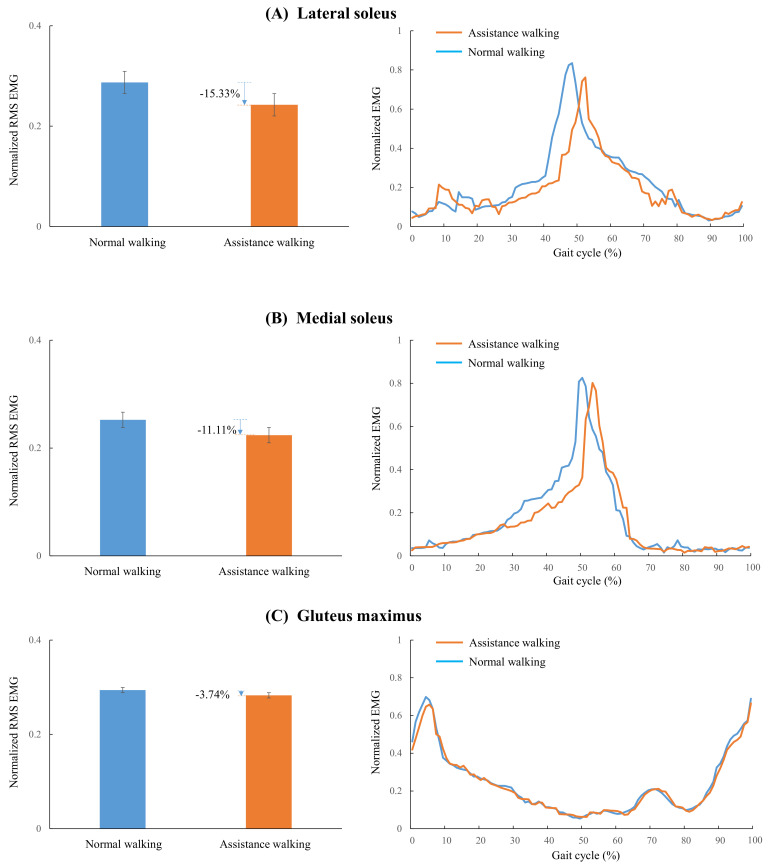
The muscle activities in assisted walking and normal walking. (**A**) Normalized RMS EMG and normalized EMG through the gait cycle of lateral soleus. (**B**) Normalized RMS EMG and normalized EMG through the gait cycle of medial soleus. (**C**) Normalized RMS EMG and normalized EMG through the gait cycle of gluteus.

The second case study was intended to compare the muscle activity of the assistance walking with that of the normal walking by EMG. Eight healthy male subjects (age 25.1 ± 5.0 years, mass 66.2 ± 9.3 kg, height 1.73 ± 0.08 m, mean ± SD) participated in the EMG measurement study. The muscle activities in normal walking and assisted walking conditions are shown in Figure 9.

The RMS value was selected as the primary metric for studying the muscle activity. The reductions in the normalized RMS EMG of the lateral soleus, medial soleus and gluteus maximus were 15.33% (*p* = 0.009, Figure 9A), 11.11% (*p* = 0.02, Figure 9B), and 3.74% (*p* = 0.356, Figure 9C), respectively. The reduction periods of the lateral, medial soleus and gluteus maximus were 27–52% (Figure 9A), 30–52% (Figure 9B), and 0–6% (Figure 9C) of a gait cycle, respectively. The normalized EMG of the lateral and medial soleus increased at about 52–60% of the gait cycle. This was likely as there was a little delay in the release of the elasticity slider of the underfoot clutch. The lateral and medial soleus overcame the remaining tension force of the spring. The EMG reduction of the gluteus was not significant. The hip extension assistance has little effect on the activity of the gluteus maximus. This was likely as the assistance force was small relative to the biological hip joint force.

### 3.5. Metabolic Cost Evaluation

Finally, we conducted metabolic cost test in unloaded walking. Six male subjects (age 25.1 ± 5.0 years, mass 65.2 ± 4.3 kg, height 1.71 ± 0.06 m, mean ± SD) were recruited to walk on the treadmill at a speed of 5 km/h.

**Figure 10:**
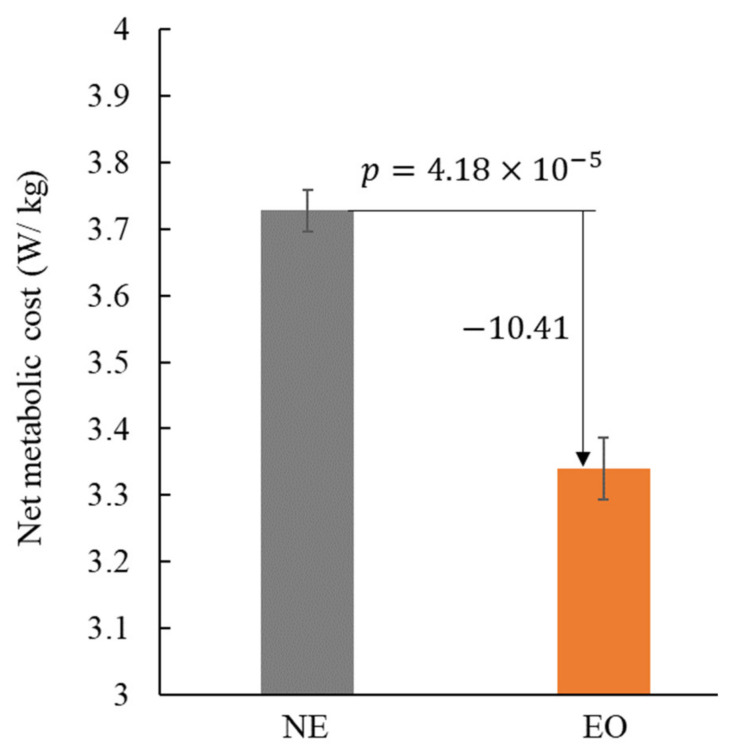
The net metabolic cost of unloaded walking reported in NE and EO conditions.

The net metabolic rates of each subject in NE and EO conditions are shown in Table 1. The net metabolic rates averaged between participants in NE and EO conditions were 3.728±0.031 W/kg and 3.340±0.047 W/kg (mean ± SEM), respectively. The metabolic cost reduction of EO walking was 10.41% compared with NE walking (refer to Figure 10). The reduction was statistically significant ($p=4.18\times 10^{-5}$).

## 4. Discussion

The comparison of popular portable LLEs is presented in Table 2. The detailed discussion is as follows:

### 4.1. Design Features

This design adapted the combination of the active and passive assistance methods instead of a single active or passive assistance mode. The design idea was to assist more motions while the system was still portable.

Previous studies have shown that some active soft exoskeletons with multi-joint assistance improved the walking efficiency compared to the transparent condition (i.e., wearing the devices with the assistance off) [11,18,29], but they did not account for the weight of the exoskeleton. On the other hand, another recent study [17] has designed a soft exosuit with single active hip extension assistance, which can provide high forces but has a weight about 1.5 times of the proposed soft exoskeleton with active-passive assistance. The lower mass of our device would facilitate its adoption among elderly and young population. Although the principle of the ankle plantarflexion assistance is similar to [22], the realization structure of this system is simpler. The ankle module in this work is suitable for wearing over the pants, while the shank interface in [22] is designed for placing under the clothes.

### 4.2. Muscle Activity

The total muscle activity reduction of the soleus including the lateral and medial soleus was 26.44%. Comparing with previous studies, the work [22] showed that the average soleus EMG was reduced by 10% when the assistance spring stiffness is 13.2 kN/m, while the optimized passive ankle assistance exoskeleton design in [21] achieved a higher reduction, equal to 22%. The muscle activity reduction of the gluteus maximus was around 3%, a little larger than the one obtained in [17]. However, the reduction was still small. The possible reason may be the tighten of the thigh wraps affected the muscle activity of the hip action muscles, and the reduction by external assistance is difficult to offset the EMG increase caused by bandage tighten.

### 4.3. Metabolic Cost

When comparing the metabolic cost reduction with previous studies, it is important to underline also the weights of the different devices. Comparing with [17], the net metabolic cost reduction obtained is higher (112%), while the system weight is reduced by 30%. These results indicate that smaller multi-joint assistance force can achieve similar walking efficiency improvement compared to larger single motion assistance force and that the hybrid assistance is also beneficial to reduce the system weight. However, comparing to the passive ankle exoskeleton presented in [21], although the weight of our device is about 3.5 times heavier, the net metabolic cost reduction increases by 45%. The walking efficiency was largely improved by the active-passive assistance. The reduction of metabolic cost is larger than the exoskeletons presented in [12,16], while the mass is smaller benefit from active-passive assistance.

### 4.4. Active Control Strategy

Both admittance control [26] and iterative learning control [17,25,30,31] strategies showed brilliant prospect in force tracking of the soft lower limb exoskeleton. The good tracking performance of the admittance control is also demonstrated by the tracking result of the proposed controller. This means that the control strategy with periodicity and flexibility may be more suitable for the control of the soft LLE. It is likely that the walking would be periodic, and the cable tension force would be flexible. The characteristics of human walking and soft robot driven determine that the control strategies with compliance are future directions.

### 4.5. Limitations of This Work

While the soft exoskeleton has shown benefit in reducing muscle activity, still it suffers from a number of limitations. The experimental tests were conducted based on the walking on a treadmill and the validation experiments only run at one speed. The walking condition when wearing the exoskeleton with assistance off was not compared. This condition should be added in following work to comprehensively test the exoskeleton.

## 5. Conclusions

In this work, a soft LLE with active hip extension assistance and passive ankle plantarflexion assistance is presented. An admittance controller based on feedforward model is found suitable to accurately track the desired active force of the hip extension. The muscle activities (the lateral soleus, medial soleus and gluteus maximus) and net metabolic cost were reduced when walking with the assistance of the soft LLE. The feasibility of the active-passive assistance to multiple lower limb motions by soft LLE was verified. In future work, the adjustment of peak time and magnitude of active hip extension assistance will be conducted to improve metabolic reduction. The optimized structure of the passive ankle exoskeleton will be designed to reduce the weight. The comprehensive evaluation in real world environments will be also conducted.

## Figures and Tables

**Table 1:** Net metabolic rate for each subject in two walking conditions.

Subject	Net Metabolic Rate (W/kg)	
	NE	EO
1	3.788	3.298
2	3.720	3.481
3	3.706	3.184
4	3.817	3.381
5	3.742	3.441
6	3.596	3.254
Mean ± SEM	3.728 ± 0.031	3.340 ± 0.047

**Table 2:** Comparison of popular portable LLEs.

Research	Weight (kg)	Net Metabolic Cost Reduction (%)	Average Muscle Activities Reduction (%)	
			Sum of Soleus	Gluteus Maximus
Panizzolo et al. [10]	0.645	3.3 ± 3.0	\	\
Mooney et al. [12]	4	8	\	\
MacLean et al. [16]	8.4	4.2	\	\
Kim et al. [17]	5.004	9.3	\	2.66
This work	3.5	10.41	26.44	3.74
Collins et al. [21]	0.816–1.006	7.2 ± 2.6	22	\
Yandell et al. [22]	0.459	\	10	\

## Data Availability

The readers can obtain data from the authors.
